# Evaluation of Atherogenic Indexes in Patients With Lichen Planopilaris: A Case-Control Study

**DOI:** 10.7759/cureus.84835

**Published:** 2025-05-26

**Authors:** Burak Celik, Merve Kaya, Gulhan A Saraç, Hakan Aksoy

**Affiliations:** 1 Internal Medicine, Cook County Health, Chicago, USA; 2 Dermatology, Health Sciences University, Ankara Bilkent City Hospital, Ankara, TUR; 3 Cardiology, Memorial Ankara Hospital, Ankara, TUR

**Keywords:** atherogenic, hair disorders, lichen planopilaris, lipids, scarring alopecia

## Abstract

Introduction: Lichen planopilaris (LPP) is a chronic cicatricial alopecia characterized by lymphocytic inflammation leading to permanent hair follicle destruction. It is associated with several systemic conditions, including hypothyroidism, dyslipidemia, hypertension, and an increased risk for cardiovascular disease. This study aims to investigate the relationship between serum lipid parameters and atherogenic indexes to evaluate the cardiovascular risk status in patients with LPP.

Methods: This retrospective study was conducted with 115 LPP patients and 115 healthy controls without LPP. Serum total cholesterol, triglycerides (TG), high-density lipoprotein (HDL), and low-density lipoprotein (LDL) were retrieved from hospital records. Atherogenic Index of Plasma (AIP), Castelli Risk Index (CRI) I and II, and Atherogenic Coefficient (AC) were calculated based on lipid profiles.

Results: LPP patients had significantly higher serum TG, total cholesterol, LDL, non-HDL cholesterol, CRI-I, CRI-II, and AC. Additionally, LPP patients were more likely to fall into the high-risk category for CRI-I, CRI-II, and AC.

Conclusions: Our study shows that patients with LPP have a higher pro-atherogenic lipid profile and atherogenic indexes. Systemic inflammation and peroxisome proliferator-activated receptor signaling dysregulation may underlie this association, necessitating closer cardiovascular and lipid monitoring in LPP patients.

## Introduction

Lichen planopilaris (LPP) is a type of cicatricial alopecia characterized by chronic lymphocytic inflammation primarily involving the isthmus of the hair follicle. This inflammation leads to permanent follicular destruction. Clinically, patients may present with symptoms such as severe pruritus, burning, follicular hyperkeratosis, follicular plugging, and perifollicular erythema, which correlate with the inflammatory activity. Typically, LPP manifests as multifocal patches with alopecia, particularly affecting the parietal scalp and forehead [1]. Although the exact pathogenesis remains unclear, the involvement of T-cells in follicular destruction suggests a possible autoimmune mechanism [2].

Numerous studies have demonstrated that LPP is associated not only with autoimmune comorbidities, such as Hashimoto's thyroiditis, systemic lupus erythematosus, and rheumatoid arthritis, but also with dyslipidemia, hypertension, increased cardiovascular risk, and metabolic syndrome [3,4].

LPP is considered an atherogenic state and is shown to be associated with dyslipidemia. To the best of our knowledge, there is no study evaluating the relationship between LPP and cardiovascular risk indexes, namely, Atherogenic Index of Plasma (AIP), Castelli Risk Index I and II (CRI-I and CRI-II), and Atherogenic Coefficient (AC). In this study, we aimed to explore the relationship between cardiovascular risk indexes and LPP [5]. AIP, CRI-I, CRI-II, and the AC are calculated based on lipid parameters measured in serum; these indexes reflect the balance between harmful and protective lipid profiles and correlate with increased cardiovascular event risk. Deranged lipid profiles secondary to LPP can lead to low-grade systemic inflammation, promoting endothelial dysfunction and increased LDL oxidation. As strong surrogates of cardiovascular risk, evaluating AIP, CRI-I, CRI-II, and AC can be a more sensitive approach to assessing cardiovascular risk in this patient group [5,6].

## Materials and methods

This retrospective study included 115 patients clinically and histopathologically diagnosed with LPP and 115 healthy controls who presented to Ankara Bilkent City Hospital Dermatology Clinic between May 2019 and September 2024. The ethical approval was obtained from the medical research ethics committee (approval number: 2-24-528). An a priori power analysis was conducted using G*Power (v3.1.9.7, Heinrich Heine University Düsseldorf, Germany) to estimate the required sample size for independent samples t-tests with an alpha of 0.05, a power of 0.95, and an effect size of d = 0.5. Healthy controls were recruited from the same dermatology clinic and screened to confirm the absence of dermatological disease via chart review and clinical examination data. Exclusion criteria were the presence of alcohol use disorder, Cushing syndrome, congenital metabolic disorders, familial hyperlipidemia, pregnancy, being in the post-menopausal period, use of oral contraceptives or lipid-lowering therapy, and chronic liver disease. Age, gender, body mass index (BMI), medical history, and laboratory values were obtained from the hospital's data system. Laboratory values included serum total cholesterol, triglyceride (TG), high-density lipoprotein cholesterol (HDL-C), and low-density lipoprotein cholesterol (LDL-C) levels.

The atherogenic indexes such as AIP, CRI-I, CRI-II, and AC were calculated using the laboratory values obtained from the medical records, according to the following formulas:

$\text{AC} = \frac{\text{NHC}}{\text{Serum HDL-C}}$

$\text{AIP} = \log \left( \frac{\text{Serum TG}}{\text{Serum HDL-C}} \right)$

$\text{CRI-I} = \frac{\text{Serum Total Cholesterol}}{\text{Serum HDL-C}}$

$\text{CRI-II} = \frac{\text{Serum LDL-C}}{\text{Serum HDL-C}}$

$\text{NHC} = \text{Serum Total Cholesterol} - \text{Serum HDL-C}$

Based on previous studies, the cutoff values of atherogenic indexes were determined to assess the cardiovascular risk. An AIP value < 0.1 was considered low, and ≥ 0.1 was regarded as high risk. CRI-I < 4, CRI-II < 3, and AC < 2 values were regarded as low risk [5,6].

Statistical analysis

The data obtained in this study were analyzed using the IBM SPSS Statistics for Windows, Version 26 (Released 2019; IBM Corp., Armonk, New York) software. Categorical variables are expressed as numbers and percentages; continuous variables are expressed as mean and standard deviation (SD). The Kolmogorov-Smirnov test was applied to test the normality of the distribution. P-values from Kolmogorov-Smirnov are as follows: HDL for patients: 0.176, controls: 0.102; LDL for patients: 0.79, controls: 0.88; TG for patients: <0.001, controls: <0.0001; and total cholesterol for patients: 0.88, controls: 0.069. An independent samples t-test was used for the comparison of normally distributed data. The Mann-Whitney U test was used for non-normal distribution, and data are expressed as median and interquartile range (IQR). Categorical variables (e.g., high-risk vs. low-risk atherogenic index, presence vs. absence of comorbidity, smoking status) were compared between groups using chi-square tests. A value of p < 0.05 was considered statistically significant.

## Results

The patient group comprised 115 individuals (55 males and 60 females), while the control group also included 115 participants (52 males and 63 females). The mean age across all participants was 38.1 years (SD: 11.2). In the patient group, five patients were on statin therapy, whereas in the control group, four patients were receiving statin therapy. Age, smoking status, and BMI did not significantly differ between patients and controls, as shown in Table 1. When examining the lipid profiles of both the patient and control groups, Table 2 demonstrates that serum TG, total cholesterol, serum LDL, and non-high-density lipoprotein cholesterol (NHC) values were significantly higher in patients with LPP compared to the control group, while serum HDL levels were not.

**Table 1:** Baseline demographic characteristics of the study population. The Student's t-test was used to compare age and BMI. The chi-square test was used to compare the number of smokers. Fischer's exact tests were used to compare chronic diseases. BMI: body mass index; DM: diabetes mellitus; CAD: coronary artery disease; SD: standard deviation

Variables	Patients (n= 115)	Controls (n=115)	p-value	t-value	χ²-value
Gender, n (%)					
Male	55 (47.8)	52 (45.2)	0.288	-	-
Female	60 (52.2)	63 (54.8)			
Age (mean±SD)	38.9±10	37.3±12.4	0.285	1.073	-
BMI (mean±SD)	26.7±4.5	27.6±5.8	0.196	-1.296	-
Smokers, n (%)	47 (40.9)	45 (39.1)	0.788	-	0.072
Chronic diseases, n (%)					
Hypertension	11 (9.5)	6 (5.2)	0.314	-	-
DM	5 (4.3)	2 (1.7)	0.446	-	-
CAD	4 (3.5)	3 (2.6)	1	-	-
Hypothyroidism	9 (7.8)	2 (1.7)	0.059	-	-

**Table 2:** Serum lipid profiles of patients and controls. The Student's t-test was used to compare serum HDL, LDL, NHC, and total cholesterol levels of the patients and controls. The Mann-Whitney U test was used to compare serum TG levels between patients and controls. For TG, the rank-biserial correlation was 0.21; Cohen's d effect sizes were 0.64 for LDL, 0.02 for HDL, 0.77 for total cholesterol, and 0.78 for non-HDL cholesterol. HDL: high-density lipoprotein; IQR: interquartile range; LDL: low-density lipoprotein; NHC: non-high-density lipoprotein cholesterol; TG: triglyceride; SD: standard deviation

Parameters	Patients (mg/dl)	Controls (mg/dl)	p-value	Normal value	t-value	Z-value
Serum TG (median (IQR))	129.7 (80)	101.31 (71)	0.001	<150 mg/dl	-	-3.234
Serum total cholesterol (mean±SD)	192.16 ± 41.52	163.72 ± 31.33	< 0.001	<200 mg/dl	5.861	-
Serum HDL (mean±SD)	50.07 ± 13.64	50.36 ± 14.96	0.879	>50 mg/dl	-0.152	-
Serum LDL (mean±SD)	117.11 ± 36.8	95.8 ± 28.89	< 0.001	<100 mg/dl	4.884	-
Serum NHC (mean±SD)	142.08 ± 41.39	113.36 ± 31.6	< 0.001	<130 mg/dl	5.914	-

Table 3 presents the atherogenic index findings for both the patient and control groups. Atherogenic risk indexes, including AIP, CRI-I, CRI-II, and AC, were significantly higher in patients with LPP compared to healthy controls.

**Table 3:** Comparison of the atherogenic risk indexes between patients and controls. The Student's t-test was used to compare the AIP and CRI-II levels of the patients and controls. The Mann-Whitney U test was used to compare the AC and CRI-I levels of patients and controls. Cohen's d=0.33 for AIP and d=0.49 for CRI-II. The rank-biserial correlation value is 0.23 for both AC and CRI-I. AC: Atherogenic Coefficient; AIP: Atherogenic Index of Plasma; CRI-I and II: Castelli Risk Index; IQR: interquartile range; SD: standard deviation

Parameters	Patients	Controls	p-value	t-value	Z-value
AC (median (IQR))	3.09 (2)	2.48 (1.55)	< 0.001	-	-3.513
AIP (mean±SD)	0.37 ± 0.29	0.28 ± 0.28	0.013	2.503	-
CRI-I (median (IQR))	4.09 (2)	3.48 (1.55)	< 0.001	-	-3.513
CRI-II (mean±SD)	2.5 ± 1	2.06 ± 0.8	< 0.001	3.680	-

According to the accepted cutoff values, atherogenic index values were categorized into high- and low-risk groups, and the difference between the case and control groups is shown in Table 4. In the chi-square analysis, patients with LPP were in the high-risk group regarding CRI-I, CRI-II, and AC indexes. Although patients were in the high-risk group compared to controls in terms of AIP values, this difference was not statistically significant (p=0.344).

**Table 4:** Comparison of the atherogenic risk index degrees between patients and controls. The chi-square test was used for all evaluations. AC: Atherogenic Coefficient; AIP: Atherogenic Index of Plasma; CRI-I and II: Castelli Risk Index; SD: standard deviation

Variables	Patients (n=115)	Controls (n=115)	p-value	χ² value
AC, n (%)				
Low risk	25 (21.7)	44 (38.3)	0.006	7.474
High risk	90 (78.3)	71 (61.7)		
AIP, n (%)				
Low risk	23 (20)	29 (25.2)	0.344	0.895
High risk	92 (80)	86 (74.8)		
CRI-I, n (%)				
Low risk	62 (54)	81 (70.4)	0.01	6.674
High risk	53 (46)	34 (29.6)		
CRI-II, n (%)				
Low risk	82 (71.3)	98 (85.2)	0.011	6.542
High risk	33 (28.7)	17 (14.8)		

Using an optimal LDL cutoff of 100 mg/dl and a high-LDL threshold of 160 mg/dl, 74 patients (64.3%) and 47 controls (40.9%) had LDL levels above the optimal limit (p < 0.001). In the high-LDL category (≥ 160 mg/dl), 11 patients (9.6%) and four healthy controls (3.5%) were identified, with no significant difference between groups (p = 0.062). Elevated TG levels were observed in 36 patients (31.3%) versus 17 controls (14.8%) (p = 0.003). Total cholesterol was elevated in 47 patients (40.9%) and 13 controls (11.3%), with a significant intergroup difference (p < 0.001). Low HDL levels were present in 59 patients (51.3%) and 63 controls (54.8%), with no significant difference between groups (p = 0.509) [7].

Age and gender were included as covariates in all multivariate linear regression models. Multivariate tests indicated that only hypertension had a significant overall effect on the combined atherogenic indexes (Pillai's trace = 0.087, F(4,217) = 5.166, p = 0.001), whereas age, BMI, gender, and diabetes mellitus showed no significant multivariate effects (all p > 0.05).

Separate general linear models for each index revealed that the full model, including age, BMI, gender, hypertension, diabetes, and their interactions, was significant for all four outcomes. For high CRI-I, the model explained 16.7% of variance (F(9,220) = 4.896, p < 0.001, R² = 0.167); for CRI-II, 11.3% (F(9,220) = 3.107, p = 0.002, R² = 0.113); for AC, 10.4% (F(9,220) = 2.836, p = 0.004, R² = 0.104); and for AIP, 10.3% (F(9,220) = 2.800, p = 0.004, R² = 0.103). Within these models, age, BMI, diabetes, and all interaction terms were non-significant (p > 0.05). However, for the AC, both gender (F(1,220) = 4.025, p = 0.046) and hypertension status (F(1,220) = 7.737, p = 0.006) emerged as independent predictors: females and participants with hypertension exhibited higher odds of elevated AC. No other predictors reached significance for CRI-I, CRI-II, or AIP.

## Discussion

LPP is an inflammatory condition classified under lymphocytic alopecias. The exact underlying mechanism of LPP is not well understood, and therapeutic options are predominantly derived from anecdotal reports and case studies [8]. The specific factors triggering the onset of inflammation in LPP remain unidentified.

Elevated levels of LDL, total cholesterol, and TG are well-established risk factors for atherogenic cardiovascular disease, cerebrovascular accidents, and metabolic syndrome [9]. AIP, CRI-I, CRI-II, and AC are validated markers developed after the Framingham Heart Study and widely used to assess atherogenic risk [5,10]. Our data indicate that patients with LPP exhibited significantly higher fasting TG, LDL, total cholesterol, and NHC levels, as well as elevated calculated values for AIP, CRI-I, CRI-II, and AC, compared to controls. In contrast, no significant difference in HDL levels was observed between the two groups.

In contrast to our findings, Conic et al. reported that patients with LPP did not exhibit higher rates of dyslipidemia compared to controls [11]. However, in this study, Conic et al. compared LPP patients with seborrheic dermatitis patients, which may be an important confounder since it was also shown that metabolic syndrome prevalence and serum TG levels are significantly higher in patients diagnosed with seborrheic dermatitis. Besides, different durations of LPP in this study may have influenced the results and may have led to an underestimation of the difference between the groups [12].

Another study involving 3,170 patients with LPP demonstrated significantly higher rates of hyperlipidemia, diabetes mellitus, metabolic syndrome, and coronary artery disease compared to the controls [4]. Nasimi et al. also indicated a higher prevalence of dyslipidemia in the LPP group compared to the controls, with 41.8% of the LPP patients having abnormalities in their lipid profiles [13]. The findings from the latter two studies are consistent with our study's findings in terms of hyperlipidemia. A potential confounding factor for the current study is the presence of hypothyroidism, which can lead to dysregulation of blood lipid profiles and is observed more frequently in patients with LPP. Hypothyroidism has been frequently associated with increased levels of TG, LDL-C, and HDL-C [14,15]. However, our assessment showed no significant differences in the prevalence of hypothyroidism between patients and healthy controls. Moreover, no significant difference was found in the number of patients with diabetes mellitus between the two groups, an element that could also be a confounding factor since DM is associated with decreased levels of HDL, increased levels of LDL-C, and postprandial hyperlipidemia [16]. These findings highlight the need for clinicians to scrutinize LPP patients for risk factors, which may require more stringent monitoring of their blood lipid profiles.

The underlying factors contributing to dysregulated serum lipid levels may be linked to the dysregulation of peroxisome proliferator-activated receptor (PPAR) signaling and systemic inflammation. PPARs, members of the nuclear receptor superfamily, play key roles in adipocyte differentiation, fatty acid oxidation, and TG metabolism [17]. Activation of PPARα in mice and humans markedly reduces TG synthesis and promotes plasma TG clearance. In addition, PPARα activation is associated with elevated plasma HDL-C levels. Activation of PPAR-γ in mice and humans is generally associated with a decrease in plasma TG [18].

Research conducted by Karnik et al. observed a reduction in the number of peroxisomes in affected and unaffected scalp tissues in patients with LPP. This observation suggests that peroxisome depletion may contribute to the inflammatory processes associated with LPP [19]. Furthermore, in murine models, PPAR-γ knockout mice have shown a predisposition to scarring alopecias, further supporting the notion that PPARs are essential in hair follicle biology [20]. PPAR-γ agonists suppress pro-inflammatory cytokines (e.g., TNF-α, IL-6) and reduce vascular inflammation, which is a key driver of atherosclerosis. PPAR-γ activation also improves endothelial function, which can help prevent plaque formation. Considering these findings, further studies are needed to investigate whether the increased lipid profile and atherogenic indexes in LPP patients are related to systemic PPAR-γ deficiency [18,20]. To substantiate these hypotheses, further research is warranted, particularly investigations evaluating the peroxisomal profiles in adipose tissue of LPP patients, which may elucidate a pre-existing derangement in peroxisome function in this patient population. Peroxisomal profile can be evaluated with skin biopsy and subsequent immunohistochemistry of peroxisomes or with quantitative methods such as PCR or microarray.

LPP shares several histopathological features with lichen planus, and these conditions may present concurrently [21]. It has been hypothesized that both LPP and lichen planus are driven by an aberrant immune response to an unidentified antigen. LPP, lichen planus, and psoriasis are considered to be driven predominantly by T-helper 1 cell-mediated inflammation [21,22]. Interleukin (IL)-17 has also been observed in LPP patients with increased expression, as reported by Dadras et al. [23]. It is thought that IL-17 enhances the production of cytokines such as IL-1, IL-6, and tumor necrosis factor-α (TNF-α). These cytokines also have important roles in LPP pathogenesis [23,24]. Elevated levels of IL-1, IL-6, and TNF-α in LPP may impair TG clearance by inhibiting lipoprotein lipase activity and reducing apoprotein-E levels, thus contributing to dyslipidemia [25].

Another potential explanation could involve a reverse-causal relationship. Previous research by Ikeda et al. demonstrated that dyslipidemia and obesity have a synergistic effect on the pathogenesis of psoriasis [26]. Similarly to psoriasis, we hypothesize that dyslipidemia may act as an accelerant in the progression of LPP. This proposition may also account for the higher prevalence of dyslipidemia observed in LPP patients compared to control groups. It has previously been reported that oxidized LDL can act as a DAMP (damage-associated molecular pattern), promoting antigen-presenting cell activation and subsequent T-cell activation in atherosclerosis [27]. Analogous to atherosclerosis, oxidized LDL can promote T-cell activation in hair follicles that may contribute to the development of LPP. Furthermore, Mailer et al. showed that a cholesterol-rich diet enhances the T-cell receptor responsiveness in CD4+ T-cells in murine models, which further supports that dyslipidemia can be a contributory factor in the pathogenesis of LPP [28].

The retrospective design and undetectable factors such as dietary habits and systemic inflammatory status were key limitations of this study. Future prospective studies with long-term follow-up and multicenter involvement are needed to better assess cardiovascular risks in patients with LPP.

## Conclusions

Our findings suggest that patients with LPP exhibit a proatherogenic lipid profile and elevated atherogenic indexes. Whether this association translates into increased cardiovascular risk warrants further investigation. Screening for dyslipidemia in LPP patients may be particularly beneficial in the presence of additional cardiovascular risk factors, such as advanced age, diabetes mellitus, or hypertension. Statin therapy should be considered when clinically indicated. While our study is limited by its retrospective design and potential unmeasured confounders, it provides a rationale for future prospective research. If a causal link is established, early lipid screening and intervention may improve long-term outcomes in this patient population.
